# Harnessing AI to fuse phenotypic signatures for drug target identification: progress in computational modeling

**DOI:** 10.1093/bib/bbag045

**Published:** 2026-02-09

**Authors:** Fengming Chen, Ranran Zhao, Xingxing Han, Huan Li, Zhishu Tang

**Affiliations:** State Key Laboratory for Quality Ensurance and Sustainable Use of Dao-di Herbs, National Resource Center for Chinese Materia Medica, China Academy of Chinese Medical Sciences, No. 16, South Xiaojie, Dongzhimen Inner, Dongcheng District, Beijing 100700 PR China; State Key Laboratory for Quality Ensurance and Sustainable Use of Dao-di Herbs, National Resource Center for Chinese Materia Medica, China Academy of Chinese Medical Sciences, No. 16, South Xiaojie, Dongzhimen Inner, Dongcheng District, Beijing 100700 PR China; Jiangsu Botanical Medicine Refinement Engineering Research Center, Nanjing University of Chinese Medicine, No. 138, Xianlin Avenue, Xianlin University Town, Nanjing 210046 PR China; State Key Laboratory for Quality Ensurance and Sustainable Use of Dao-di Herbs, National Resource Center for Chinese Materia Medica, China Academy of Chinese Medical Sciences, No. 16, South Xiaojie, Dongzhimen Inner, Dongcheng District, Beijing 100700 PR China; School of Chinese Materia Medica, Beijing University of Chinese Medicine, No.11, North Third Ring Road East, Chaoyang District, Beijing 100029, PR China

**Keywords:** drug-induced gene expression profiling, drug-target interaction prediction, phenotype-based prediction, biological network models, association analysis

## Abstract

Computational models integrating large-scale gene expression profiles provide a powerful approach for predicting multi-target drug interactions (DTIs). Unlike traditional experimental and computational methods that often require detailed structural or target-specific information, gene expression-based models leverage reference transcriptional signatures. This enables functional inference of interactions without explicit structural data, offering a valuable strategy in data-limited scenarios. By incorporating phenotypic information, these models bridge phenotype screening and target prediction, establishing a novel paradigm for target identification. This review introduces and compares current target identification methods, emphasizing the unique advantages of gene expression profiling in DTI prediction. We also outline major public databases and their applications. As an effective hypothesis-generation tools, computational DTI models reduce experimental costs, enhance understanding of multi-target mechanisms, and accelerate drug discovery. We categorize and analyze three primary model types utilizing large-scale gene expression data: biological network-based, association-based, and multimodal integration approaches, discussing their respective strengths and limitations. Key challenges and future directions are also addressed, including data integration, algorithm optimization, and multi-omics fusion, to fully realize the potential of gene expression data in multi-target drug prediction. This review offers comprehensive guidance on advanced tools, databases, and methodologies, enabling novel research paths for unbiased multi-target exploration. By linking phenotype screening with computational analysis, this integrative approach is expected to advance precision medicine, especially in uncovering drug mechanisms in complex diseases, offering promising prospects.

## Introduction

Target identification, particularly for compounds eliciting multi-target effects (activity at multiple intended targets), is crucial for evaluating on-target activity and detecting potentially harmful off-target effects (interactions with unintended targets) associated with toxicity [1]. Advances in drug target knowledge have led to significant recognition of systemic pharmacology (one drug–multiple targets) [2–4]. Single-target ligands often prove inadequate for treating complex diseases like cancer, neurological disorders, and metabolic diseases, which often involve intricate mechanisms [5–9]. Consequently, phenotypic screening has regained emphasis. Unlike traditional target-based approaches like molecular bioassays (which measure ligand binding affinity to a specific protein), phenotypic profiling techniques (e.g. gene expression signatures, cell painting) now offer high-throughput, accessible, and cost-effective capabilities, enabling their large-scale application in chemical biology and drug discovery. Computational methods leveraging this data facilitate efficient active compound screening and can predict unknown or unexpected outcomes [10–15]. Thus, integration of target-based and phenotype-based methods has reemerged as an effective drug discovery model [16, 17].

Compared to other phenotypic information like cell painting, drug perturbation transcriptomic data offers a more comprehensive, high-resolution snapshot of a drug’s molecular impact on gene expression networks. Furthermore, other omics analyses (e.g. proteomics, metabolomics) currently lack scalability to millions of compounds [18–20]. This detailed molecular insight facilitates hypothesis-free identification of potential drug targets and mechanisms, surpassing traditional phenotypic methods capturing limited cellular changes [21]. Discovering new drug-target interactions is essential for identifying new targets for existing drugs (e.g. imatinib, sildenafil) [22, 23] and developing drugs for disease-specific genes. Thus, predicting target proteins for drug candidates is a highly motivated endeavor. Using drug-induced gene expression profiles for phenotype screening circumvents limitations of experimental methods like chemical proteomics (e.g. challenges with molecular labeling and protein deciphering) and purely computational models (e.g. activity cliffs and high computational demands) [24, 25]. This approach holds potential in new drug discovery [26–29], drug repositioning [30–32], adverse events prediction [33, 34], and precision medicine [35–39]. Advancements in high-throughput sequencing and single-cell transcriptomics enable more rapid, comprehensive, and cost-effective access to condition-specific gene expression changes [40–44], thereby greatly enhancing research applications. As data volumes grow, computational analysis using drug-induced gene expression profiling will become increasingly effective at uncovering multi-target and off-target effects of compounds, accelerating drug development.

This review comprehensively examines computational methods for drug target prediction that primarily utilize drug perturbation transcriptomic data. It synthesizes recent research advances, core challenges, and future directions within the field. Focusing on perturbation transcriptomics as a dynamic functional data type, the review discusses key prerequisites for its practical translation, including data quality, standardization protocols, and inherent sampling biases, such as the limited cell line coverage in common perturbation databases. The article systematically categorizes the methodological landscape, ranging from comparative analysis to advanced deep learning and multi-modal integration models, and provides a critical commentary on the principles and applications of different modeling paradigms, including network analysis and machine learning (ML) approaches. Furthermore, it explores modeling strategies that integrate multi-source data to enhance prediction robustness and overcome the limitations of single data types. Finally, the review outlines potential pathways to address current bottlenecks, such as the integration of multi-modal biological knowledge, the development of robust data processing pipelines, and the advancement of interpretable models. It is hoped that this work will offer a clear technical reference for researchers in computational biology and drug discovery, thereby facilitating the development of next-generation transcriptomics-driven target identification tools.

## Methods for drug target identification and comparison

**Figure 1:**
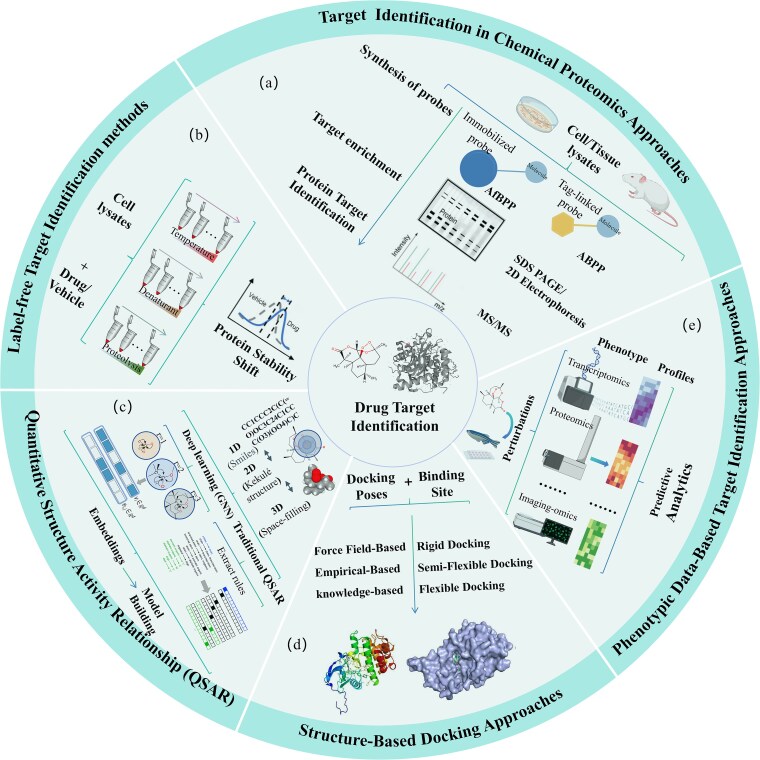
Overview of drug target identification techniques. (a) Chemical proteomics workflow: Involves probe design, target enrichment, and protein identification, using either AfBPP, where bioactive molecules capture targets via resin binding, or ABPP, employing labeled probes to flexibly capture drug-target interactions. (b) Non-labeled target identification: Detects DTIs by assessing protein stability changes under various experimental conditions (e.g. temperature shifts, protease degradation). (c) QSAR methods: Utilize molecular descriptors across structural levels (1D–3D); deep learning advancements now enable dynamic embedding for enhanced prediction accuracy. (d) Molecular docking: Uses a “key-lock” approach to predict ligand-receptor binding modes and sites. (e) Phenotype-based target identification: Combines experimental and computational analyses to capture a broad range of biological effects, from cell morphology changes to gene expression regulation.

Target identification is essential in drug discovery, particularly when addressing complex multi-target drug interactions. Methodological strategies span biomolecule-interaction-centric approaches (e.g. chemical proteomics, label-free techniques) and data-driven prediction approaches (e.g. QSAR, network modeling) [45–47] (as shown in Fig. 1). Over the past decade, interaction-centric techniques like chemical proteomics and label-free techniques have played a pivotal role in drug target identification by directly detecting physical interactions between small molecules and proteins [48, 49]. Meanwhile, data-driven prediction approaches such as QSAR, molecular docking, and network-based modeling have gained increasing relevance, especially as high-throughput data resources have expanded. Additionally, models incorporating phenotypic information offer a powerful complement by integrating large-scale data for target prediction [41, 50]. This section introduces and compares these methods, discussing their applicability, advantages, and limitations in drug target prediction, as summarized in Table 1.

**Table 1:** Comparison of different methods for drug target identification.

Methods	QSAR	Molecular docking	Phenotype-based prediction	Label-based methods	Label-free methods
Core data type	Chemical structure, properties	3D structure of molecules and targets	Gene expression, cellular images, protein expression, etc.	Molecular probes (fluorescent, radioactive) for tracking binding	Biophysical properties like solubility, oxidation rate, and protease sensitivity
Strategy	Predict biological activity from chemical structure	Simulate molecular binding to protein targets	Analyze gene expression or phenotypic data (e.g. cell images) to infer mechanisms	Specific target identification via labeled probes	Identify targets through changes in protein stability under varying conditions
Structure-based	Yes	Yes	No	Yes (relies on labeled probes)	No
Target-based	No	Yes	No	Yes	Yes
Advantages	Rapid screening, time and cost-efficient	Precise binding prediction, supports drug design	Captures systemic effects, suitable for multi-target discovery and repositioning	High specificity, precise tracking, suitable for known targets	No chemical labeling required, suitable for complex compounds and high-throughput screening
Limitations	Activity cliffs, cannot directly reveal molecular mechanisms	Requires high-quality protein structure, computationally intensive	Complex data, requires high-quality input and advanced tools	Labeling may alter chemical properties, limited in complex molecules	Limited sensitivity for low-abundance targets, reliant on specific conditions, may miss biological network complexity
Representative tools	MolPMoFiT, CoMSIA	AutoDock, VirtualFlow Ants	CMap, KGDRP	Fluorescent probes, PAL	CETSA, DARTS, SPROX, SIP

### Biochemical techniques: label-based and label-free approaches

Biochemical strategies for target identification encompass label-based chemical proteomics and label-free methods that detect protein stability or migration shifts upon ligand binding (Fig. 1a). These approaches, which prioritize the direct physical measurement of biomolecular interactions, form a cornerstone of drug discovery. Currently, chemical labeling methods are more prevalent in research [51]. Innovations like click chemistry and photoaffinity labeling (PAL) have enhanced specificity [52, 53]. Despite its utility, chemical proteomics faces challenges including nonspecific binding, the need for carefully balanced probe design, limited proteome coverage for probe-less proteins, and the demand for robust computational tools to integrate data with other omics datasets.

Label-free techniques identify drug targets by measuring ligand-induced changes in protein stability under perturbations like temperature shifts or chemical denaturation (Fig. 1b). These methods do not require structural modification of the drug. Key techniques include Cellular Thermal Shift Assay (CETSA) [54], and Drug Affinity Responsive Target Stability (DARTS) [55]. They are particularly advantageous for studying complex molecules (e.g. natural products) or low-abundance targets. High-throughput adaptations enable efficient screening of potential targets in complex biological samples like cell lysates. Challenges persist, however, in detecting low-abundance or low-specificity proteins due to limited sensitivity and mass spectrometry coverage. Experimental conditions such as pH and ionic strength can also significantly influence results, adding complexity to target identification. Additionally, emerging strategies like genomic library screening [56], protein degradation [57], sequencing of drug-resistant mutants [58], and organoid modeling [59] are advancing the field but fall outside the scope of this detailed discussion.

### Structure data-driven approaches for target identification

Driven by expanding high-throughput data and ML, data-driven approaches have gained prominence [60]. These methods span a range of techniques, including structure-based approaches (e.g. molecular docking), which rely on known or predicted 3D protein structures to simulate binding events; ligand-based models, which infer targets based on the similarity of biological activity profiles among compounds. Deep learning has further transformed the field by enabling models to learn dynamic molecular embeddings directly from SMILES strings or molecular graphs (Fig. 1c), leading to more accurate bioactivity predictions [61]. Pre-training and transfer learning methods have substantially improved QSAR modeling. For example, MolPMoFiT [62] uses self-supervised pre-training on millions of unlabeled ChEMBL molecules, followed by fine-tuning, which greatly enhances cross-task generalization. Concurrently, deep molecular docking, combining deep learning with traditional methods, has improved the efficiency and accuracy of large-scale virtual screening [63]. This includes developing QSAR-style models for rapid binding score prediction, enabling high-throughput screening of ultra-large libraries. A representative platform, VirtualFlow Ants [64], integrates multiple docking programs and scoring functions, and significantly boosts screening precision. However, structure-based descriptors have inherent limitations. Their predictive performance depends on the quantity and quality of activity data, and models are limited by their training-set-defined applicability domain. Moreover, minor structural changes can cause large activity shifts, a phenomenon known as the “activity cliff.” While no approved drug has been discovered solely through QSAR, growing evidence shows these methods, especially combined with deep learning, can significantly accelerate early-stage small-molecule drug candidate discovery [61, 65].

### Phenotypic data-driven target prediction

Phenotypes encompass diverse biological effects across organismal and cellular levels, including disease symptoms, changes in cell morphology, regulation of gene and protein expression, and alterations in metabolic pathways [12, 66]. Unlike traditional experimental or computational target identification methods that rely on small molecule structures (e.g. chemical labeling or molecular docking), phenotype-based target identification combines experimental observations with computational analysis [67–69]. By systematically observing compound-induced biological effects and applying computational analysis (Fig. 1e), this approach enables more accurate target prediction. Historically, target-based and phenotype-based drug discovery have been considered separate approaches [70]. However, computational target prediction methods based on phenotypic data bridge this gap, combining the strengths of both methods. This integration offers a comprehensive understanding of drug effects at the system level and provides precise predictions of specific molecular targets [71, 72].

Phenotype-based methods hold particular promise for target deconvolution in complex systems such as natural products [73, 74]. They can infer mechanisms of action without requiring prior detailed target information, opening new avenues in drug discovery [75]. For instance, even with chemically complex traditional medicine formulations or plant extracts of unclear active constituents, phenotypic data allow systematic assessment of biological effects. Phenotype-based techniques can analyze the biological mechanisms of these complex substances, revealing novel targets and signaling pathways [76–80]. Common phenotypic screening strategies, including chemical genetics, transcriptomic analysis, and cell morphology comparison, collectively capture multidimensional drug effects on biological systems. Advances in high-throughput technologies and big data analytics now enable comprehensive capture of global phenotypic changes induced by compounds [81]. Integrating these data-rich phenotypic observations with computational models enhances target prediction accuracy and significantly improves drug screening efficiency, accelerating discovery and offering potential for treating complex diseases and developing multi-target drugs [13]. For example, knowledge-guided heterogeneous graph neural networks such as KGDRP [82] can integrate phenotypic data, gene expression profiles, and chemical structures, improving both drug response prediction and target/pathway identification. However, challenges remain. Limited drug overlap across different data sources can lead to cold-start problems, constraining relationship-dependent models like graph neural networks. Furthermore, the high heterogeneity of biomedical data can create high-dimensional feature spaces upon direct integration, increasing computational cost and overfitting risk. Developing computational methods that can effectively align, reduce dimensionality, and fuse multi-source phenotypic data therefore remains a key challenge.

### Other computational methods

Emerging computational approaches, including network analysis, are gaining attention for drug target prediction. These methods construct biomolecular networks, such as drug–target, drug–drug, and protein–protein interaction networks (PPINs), where nodes represent drugs or proteins, and edges denote their interactions or functional associations [83]. Though still evolving, network analysis offers a valuable perspective by revealing potential key targets and their relational context. ML and deep learning techniques are also increasingly applied, particularly for processing large-scale chemical genomic data and capturing nonlinear relationships [84, 85]. Models such as DeepDTI [86] and PPAEDTI [87] have improved prediction accuracy by framing DTI prediction as a binary classification task and integrating molecular structural features with graph networks. While still in early research stages, their predictive power is becoming more recognized. Additionally, text mining using natural language processing (NLP) has been introduced to automatically extract potential targets and interactions from scientific literature [88, 89]. This approach supplements limited experimental data, although it requires further validation and optimization.

## Drug perturbation transcriptomics for target prediction: advances, resources, and applications

Gene expression profiling is one of the highest throughput, lowest cost, and high-dimensional analysis data types currently used for phenotype-based predictive analysis [90]. Especially in the post-genomic era, rapid advancements in high-throughput technology have revolutionized drug discovery, allowing scientists to better understand disease-related biological systems. Specifically, drug perturbation transcriptomic data, representing comprehensive gene expression changes induced by compounds in cells, tissues, animals, and humans, are essential in drug target identification [41].

### Key advances behind the success

The advantages of phenotypic information in target prediction lie in its ability to comprehensively capture an organism’s response to a drug, applicability to novel/complex compounds, and integrate experimental observations with computational analysis. This approach bypasses limitations of structure-based methods (e.g. labeling, docking), requires no prior target knowledge, and identifies potential targets without enrichment. In contrast, scaling non-transcriptomic resources remains challenging. These data are often experiment-specific, such as proteomics studies that focus on particular phosphorylation sites, which are costly to scale. Additionally, differences in experimental targets and methods hinder direct data comparison and integration across laboratories, limiting their broader applicability. While transcriptomic data also pose integration challenges [91, 92], they offer high throughput, low cost, and extensive coverage of gene expression patterns under diverse conditions. This data type reflects complex biological effects and is well-suited for integration with biological network resources, making it ideal for large-scale analysis and pattern recognition.

Widespread application stems from two advances: (i) high-throughput transcriptomics platforms, such as L1000 [41], HTS^2^ (high throughput sequencing based high throughput screening) [93], DRUG-seq (digital RNA with pertUrbation of genes sequencing) [43], enable simultaneous analysis of thousands of genes across numerous samples. This allows for detection of subtle but significant regulatory trends, building a solid data foundation for target prediction. (ii) Advances in deep learning and other algorithms have significantly enhanced complex data processing capabilities [94]. These algorithms address signal masking in genome-wide expression profiles by accurately identifying gene feature patterns, enabling more precise signal detection and minimizing interference from biological pathway noise. Tools such as Connectivity Map (CMap) leverage these algorithms to capture gene expression changes induced by drugs, offering robust support for target prediction [95]. These tools lower the threshold of complex data analysis, enabling even researchers who lack a background in bioinformatics or computational biology to perform effective target prediction [96].

While these algorithmic advancements have improved the handling of gene expression data complexity, limitations remain, particularly in capturing protein-level changes and dynamic biological processes [97]. To address these, biological network analysis serves as an important complementary tool. Integrating multi-level network data [e.g. protein interaction and gene regulatory networks (GRNs)] provides upstream and downstream relational insights that enhance the accuracy and biological interpretability of target prediction [35]. Models based on gene expression profiles complement traditional target prediction approaches. For example, models using high-throughput phenotypic fingerprints (HTSFP) perform comparably or slightly better than those using traditional molecular fingerprints (ECFP) [98]. Importantly, they identify active molecules with distinct, non-overlapping chemical structures. This highlights the unique value of HTSFP in enabling scaffold hopping. These findings suggest that transcriptomic data not only overcome the limitations of traditional target identification methods but also provide robust support and wider applicability in target prediction.

### Databases and case applications

Rapid transcriptomics development over two decades has spurred a surge in specialized databases, with over 200 resources now providing extensive gene expression data. Among them, the CMap project stands as a milestone in drug-induced transcriptomics, expanding from 564 profiles in 2006 to 1.3 million in 2017 [41, 95]. This expansion reflects significant progress in data generation, providing unprecedented support for drug discovery, mechanism-of-action analysis, and drug repurposing. Our analysis focuses on databases containing drug-induced transcriptional profiles (Table 2), which can be divided into two main categories based on data origin and construction: Integrated and Generated databases. Integrated databases compile data from multiple sources, often covering gene expression profiles from various laboratories and experimental conditions. For example, the NCBI GEO [99] database faces challenges in effectively integrating multi-source data and addressing batch effects. Generated databases, in contrast, are produced by single or specific research projects, ensuring consistent experimental conditions and data quality. Examples such as CMap [95] offer standardized experimental design and high consistency, making them particularly valuable for targeted biological studies. This categorization provides insight into the unique strengths and applications of each database type across different research scenarios.

**Table 2:** Summary of databases for drug-induced gene expression profiles across species and platforms.

Resource	Perturbation type(s) (num)	Category	Species	Platforms	Access
CMap v1 [95]	Small molecules (164)	GDB	Human	Affymetrix Microarrays	https://clue.io/
CMap v2/ LINCS [41]	Small molecules (19 811), shRNA (18 493), cDNA (3462), biologics (314)	GDB	Human	L1000	https://lincs.hms.harvard.edu/
CRCGN [106]	Small molecules (330)	GDB	Human	L1000	https://carcinogenome.org/
NCBI GEO [99]	>200 000 Studies/DataSets, Small molecules (unknown)	IDB	Multiple Organisms	Microarray, RNA-Seq, ChIP-Seq, Other HTS Platforms	https://www.ncbi.nlm.nih.gov/geo/
ArrayExpress [107]	Small molecules (1556)	IDB	Multiple Organisms	Microarray, RNA-Seq, ChIP-Seq, Other HTS Platforms	https://www.ebi.ac.uk/arrayexpress
Expression Atlas [108]	>4424 datasets, Small molecules (unknown)	IDB	Multiple Organisms	Microarray, RNA-Seq, ChIP-Seq, Other HTS Platforms	https://www.ebi.ac.uk/gxa/home
DeMAND [109]	FDA approved compounds (92)	GDB	Human	Microarray	https://www.ncbi.nlm.nih.gov/geo/query/acc.cgi?acc=GSE60408
Open TG-GATEs [110]	Small molecules (170)	GDB	rat, Human	Microarray	https://toxico.nibiohn.go.jp/english/index.html
Fish Cmap [111]	Small molecules (around 140)	GDB	zebrafish	Microarray	GSE38070, GSE60202, GSE70807, and GSE70936
CREEDS [112]	Small molecules (343), gene (1186)	IDB	Human, Mouse or Rat	Microarray, RNA-Seq, ChIP-Seq, Other HTS Platforms	https://maayanlab.cloud/CREEDS/
MSigDB [113]	Chemical and genetic perturbations (3494)	IDB	Human, Mouse or Rat	Microarray, RNA-Seq, ChIP-Seq, Other HTS Platforms	https://www.gsea-msigdb.org/gsea/msigdb/human/collections.jsp#C2
CDS-DB [114]	Small molecules (85)	IDB	Human clinical	Microarray, RNA-Seq	http://cdsdb.ncpsb.org.cn/
DrugSig [115]	Small molecules (1300)	IDB	Human, Mouse or Rat	Microarray, RNA-Seq	http://biotechlab.fudan.edu.cn/database/drugsig/
DRUG-seq [43]	Small molecules (433)	GDB	Human, Mouse	RNA-Seq, DRUG-seq	https://www.ncbi.nlm.nih.gov/geo/query/acc.cgi?acc=GSE120222
DrugMatrix [116]	Small molecules (600)	GDB	Rat	Microarray	https://www.ncbi.nlm.nih.gov/geo/query/acc.cgi?acc=GSE59927
ChemPert [117]	Small molecules (2566)	IDB	Human, Mouse and Rat	Microarray, RNA-Seq, ChIP-Seq, Other HTS Platforms	https://chempert.uni.lu/
HERB [118]	Herbs (20), Small molecules (152)	IDB	Human, Mouse and Rat	Microarray, RNA-Seq, ChIP-Seq, Other HTS Platforms	http://herb.ac.cn/
TMNP [119]	Herbs (40)	IDB	Human, Mouse and Rat	Microarray, RNA-Seq, ChIP-Seq, Other HTS Platforms	http://www.bcxnfz.top/TMNP/
TCM Hub [120]	Small molecules (102)	IDB	Human	RNA-Seq	http://tanlab.ucdenver.edu/TCMHub
ITCM [121]	Small molecules (496)	GDB	Human	RNA-Seq	http://itcm.biotcm.net

Abbreviations: Integrative Database, IDB; Generative Database, GDB.

This strategy has been widely applied to predict novel targets or functions for diverse chemicals. For instance, Chen *et al.* [14] identified hyperforin as a lead anti-obesity compound by revealing dihydrolipoamide S-acetyltransferase as its direct target. Lim *et al.* [100] discovered a novel target for a urinary calcitonin-II receptor antagonist by correlating its expression profile with other drugs. Özdemir *et al.* [101] employed CMap analysis to pinpoint Rho kinase as a target of antidiabetic drugs metformin and tolbutamide. Notably, this approach continues to drive discovery in the most recent research. In oncology, Cho *et al.* performed a signature similarity search against the LINCS L1000 database using a transcriptomic signature from CBP20 knockdown, successfully repositioning raloxifene, purpurogallin, and enoxacin as potential anticancer agents that mimic CBP20 loss-of-function [102]. For Chronic Kidney Disease (CKD), Xiao *et al.* constructed a tubulointerstitial fibrosis gene signature from human CKD transcriptomes and screened the LINCS L1000 library to identify narciclasine as a top candidate for reversing this signature, with efficacy validated in multiple preclinical kidney injury models [103]. Beyond direct signature matching, advanced computational models are refining target deconvolution. Liu *et al.* utilized a graph convolutional network-based algorithm to predict the cGAS-STING pathway as the target of Ginkgetin in alleviating cellular senescence and inflammation, later confirmed experimentally [104]. Similarly, Sun *et al.* combined graph convolutional network prediction with biophysical assays to identify Gli1 as a direct target of berberine, elucidating its novel mechanism of inducing ferroptosis in colorectal cancer [105].

### Strategies for mitigating data heterogeneity and sampling bias

To enable systematic comparison, we summarize representative ML methods from recent studies evaluated on shared benchmark datasets. Table 3 presents key performance metrics of these models on LINCS L1000, providing an intuitive reference. However, because studies differ in data selection, preprocessing, negative sampling, and evaluation protocols, results should be interpreted within their specific experimental contexts. Most expression-based supervised models are trained primarily on relatively homogeneous cell line perturbation data from LINCS. While LINCS L1000 contains approximately 1.3 million perturbation profiles across more than 70 human cell lines, the data distribution is highly imbalanced: a small number of cell lines (e.g. MCF7, PC3) dominate the dataset, and only ~12% of cell lines have sufficient coverage for large-scale pharmacogenomic analysis [122]. This imbalance, together with cell line-dependent transcriptional responses to compounds, limits the biological representativeness of the training data. As a result, model performance may be inflated on benchmark datasets while generalization to novel cell types or perturbation settings remains limited.

**Table 3:** Comparative overview of DTI prediction models utilizing LINCS L1000 transcriptional perturbation data.

Model	Core method	Key data (LINCS L1000)	Key metric	Data split	Negative sampling
SSGCN [133]	Dual spectral GCNs comparing compound & gene perturbation profiles.	Phase I & II, Lvl 5; ~2 K compounds.	HR@100 = 0.53, AUPRC = 0.84	Scaffold-based 8:1:1	Random pairs (3:1 ratio)
SVM; MLP; NB; KNN [134]	Multiple ML models trained on expression profiles.	Phase I & II, Lvl 5; PC3 & MCF7 cells; 765 compounds.	AUROC = 0.73; 0.66; 0.65; 0.60	20% holdout +5-fold CV	Balanced random selection
DeepCodex [135]	Deep densely connected network for profile embedding.	Lvl 4; PC3 & MCF7 cells; 411 compounds.	AUROC = 0.906	80/20 train/test	Random subsampling (10 K)
DNN-PM [136]	DNN concatenating drug & target embeddings.	Lvl 5; 2494 compounds.	AUROC = 0.849	10-fold CV	Balanced random (1:1)
Filzen et al [137].	Deep autoencoder for replicate-aware binary barcodes.	PC3 & ME180 cells; 3699 compounds.	F1 = 0.87	8:1:1 train/val/test	Random pairs (2x)
DrSim [138]	PCA & LDA for transcriptional similarity.	11 cell lines; 2597 compounds.	Accuracy = 0.38	Unsupervised	Unsupervised
FRoGS [139]	Functional gene embeddings (GO annotations) for signature similarity.	Lvl 4; 1438 compounds.	Recall@5% = 0.36	Compound-based 5-fold CV	Random (1:99), weighted to 1:1
BANDIT [140]	Bayesian fusion of multiple drug similarity types.	CMap (1309 compounds)	AUROC = 0.89	5-fold CV (drug pairs)	Non-shared-target pairs
MoAble [141]	Maps compound structures to signature-like embeddings.	Lvl 5; ~20.9 K compounds.	AUROC = 0.642	Compound-wise 70/15/15 split	Triplet loss (random compounds)
MDTips [142]	Multimodal deep learning fusing KG, structure, and expression.	Lvl 5 data.	AUPRC = 0.951, AUROC = 0.970	10-fold CV	Undersampled (1:2 ratio)
GraphDTI [143]	Integrates multi-source features via encoding and MLP.	Lvl 5; 30 cell lines; 462 compounds.	AUROC = 0.999; 0.996	10-fold CV (random/cluster)	Balanced down-sampling (1:1)
Moshkov *et al.* [18]	Late fusion of chemical, morphological & expression features.	U2OS cells; ~16.2 K compounds.	Accuracy = 0.21	5-fold CV (scaffold-based)	Balanced per compound (1:1)
FMBS [144]	Bayesian & voting integration of 25 biological signatures.	1150 compounds.	AUROC = 0.92	5-fold CV (8:2 split)	Non-shared-target pairs
PertKGE [145]	Multi-level knowledge graph embedding for perturbations.	Phase I & II, Lvl 5; PC3 cells; ~10.9 K compounds.	HR@10 = 0.266	5-fold CV (by compound)	Corrupted triples
SETComp [146]	Set-based deep learning & transfer learning for cell-specific effects.	LINCS L1000 data.	AUROC = 0.948	Pre-train: 7:3 train/val	Down-sampled balanced classes

Notes: Data “Level” refers to the processing stage of the LINCS data. AUROC, Area Under the Receiver Operating Characteristic Curve; AUPRC, Area Under the Precision-Recall Curve; CV, Cross-Validation; GO, Gene Ontology; HR@K, Hit Rate at rank K; KG, Knowledge Graph; LDA, Linear Discriminant Analysis; MLP, Multilayer Perceptron; MoA, Mechanism of Action; PCA, Principal Component Analysis; PPI, Protein–Protein Interaction.

Data preprocessing and normalization are therefore critical. Batch effects and platform differences substantially affect data comparability [123]. Without correction, only a small fraction of compounds shows consistent signals across databases [124]. Statistical normalization methods (e.g. z-score, TPM) and batch correction techniques such as mean centering and synthetic profile construction have been shown to improve data quality and model robustness [125, 126]. In addition, explicit evaluation of cross-cell-line and cross-dataset generalization should be incorporated into standard validation pipelines, as ~43% of perturbations exhibit strong cell-type specificity [127].

To more fundamentally address data scarcity and heterogeneity, emerging computational strategies offer promising solutions. Deep generative models and pre-training approaches can extend data utility by learning perturbation-conditioned transcriptional responses. For example, PRnet integrates untreated expression profiles with compound features using denoising autoencoders to predict responses for novel compounds at both bulk and single-cell levels [128]. Similarly, the “virtual cell” model STATE [129], pre-trained on 267 million single cells using a bidirectional Transformer, captures cellular heterogeneity and predicts responses to drug or genetic perturbations without explicit distributional assumptions. In parallel, architectural innovations that integrate biological prior knowledge [130], such as graph neural networks with global attention (e.g. BulkFormer [131]) or network-aware graph learning methods (e.g. scGraphDap [132]), enable more robust representation learning across heterogeneous transcriptomic datasets. Together, these advances suggest that future progress will depend not only on larger datasets but also on computational frameworks that more effectively integrate data and biological knowledge.

## Computational strategies for target identification using large-scale drug-induced transcriptomic data

Multiple drug-induced gene expression databases offer valuable opportunities while posing challenges for effectively mining large-scale data for target identification [147]. Selecting and applying appropriate strategies requires systematic guidance. This review provides practical guidelines by linking expression data with analytical strategies. In the following sections, we systematically review key approaches for drug target prediction using gene expression profiling data, summarizing three core strategies: biological network integration, expression-based association analysis, and multimodal data fusion. These methods illustrate how transcriptomic data can be effectively integrated with computational techniques to enhance drug target identification accuracy and deepen our understanding of their mechanisms of action [148].

### Integration of gene expression profiles with biological networks

Gene expression profiling captures genome-wide responses but struggles to distinguish direct targets from downstream effects using basic enrichment methods (e.g. KEGG, GSEA). Network-based approaches like GRNs and PPINs address this limitation [149]. Their integration enables systematic understanding of drug actions, isolating direct targets, and improving predictive accuracy.

#### Gene regulatory network-based target identification

Despite revealing genome-wide expression changes, distinguishing direct drug targets from indirect effects remains challenging. GRNs regulate cellular processes through interactions between transcription factors, genes, and downstream targets [150]. Understanding these regulatory mechanisms is essential for drug target prediction and elucidating disease mechanisms [151]. GRN inference aims to reconstruct GRNs by inferring regulatory relationships among genes from expression data [152]. The advent of high-throughput technologies, like RNA sequencing and single-cell transcriptomics, has expanded our capacity to analyze gene interactions. However, GRN inference involves complex, multi-level interactions—linear and non-linear, functional and non-functional—that complicate the process. Moreover, gene regulation is often not immediate; it includes delays due to transcription factor production and protein regulation [153]. Combining gene expression data with GRNs offers promising potential in drug target prediction but faces significant challenges related to modeling the dynamic nature of gene regulation. Specifically, inferring accurate GRNs is complicated by the need to capture temporal changes and dynamic interactions, which static network models and steady-state assumptions fail to represent adequately [154–156].

**Figure 2:**
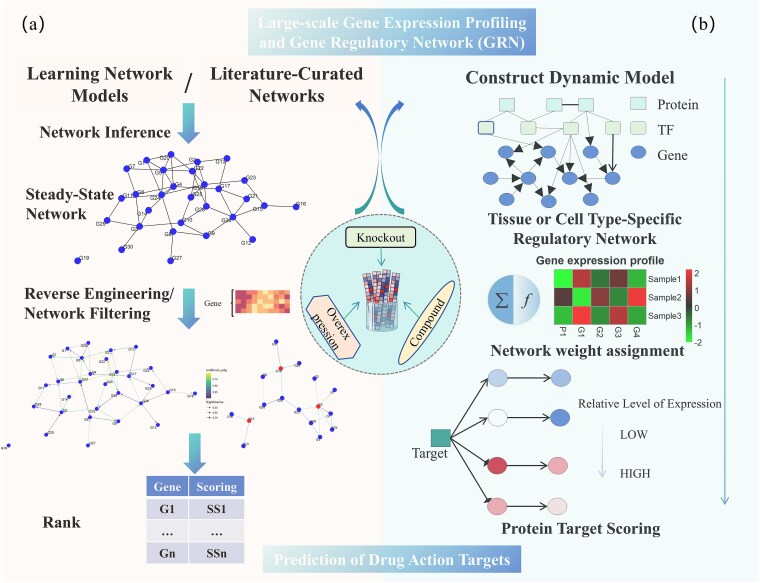
Target prediction methods integrating gene expression profiles with GRNs. (a) Static GRNs: Early target identification methods assumed unchanging gene regulatory relationships before and after drug treatment, relying on static GRNs to evaluate drug-induced expression changes. (b) Dynamic modeling: Advanced methods like ProTINA integrate dynamic modeling with GRN topology to capture time-dependent regulatory changes. Combines PGRN models with steady-state and time-series data, using ODE modeling to infer drug effects on gene activation and pathway inhibition, effectively predicting drug targets even when changes in target gene expression are minimal.

Early studies on target identification based on drug-induced expression profiles often relied on static GRNs (Fig. 2a), assuming unchanging gene regulatory relationships pre- and post-drug treatment [157]. For instance, Gardner *et al.* [158] developed the Network Identification by Multiple Regression (NIR) method, which combines steady-state gene expression data with linear regression to infer GRNs from large-scale perturbation data, identifying direct drug targets. Subsequent methods like Mode of Action by Network Identification [159], Sparse Simultaneous Equation Models [160], and DEMAND [109] evolved from NIR and employ network filtering techniques to identify target genes and their regulatory pathways. Despite advancements, challenges persist, as GRN inference remains an underdetermined problem due to limited experimental data. Additionally, these methods often rely on static network structures, which overlook dynamic regulatory changes across conditions, thereby limiting target prediction accuracy.

To overcome these limitations, dynamic modeling with time-series data has been introduced (Fig. 2b). DeltaNet offers a novel solution by using Ordinary Differential Equation (ODE) modeling, enabling direct target prediction from data while inferring GRNs implicitly [161]. Combining LASSO regularization and Least-Angle Regression, DeltaNet addresses the “dimensionality catastrophe” in gene expression data without complex parameter tuning. DeltaNet’s enhanced version, DeltaNeTS+, integrates steady-state and time-series data with existing GRN structural information, further improving prediction accuracy [162]. This dynamic modeling framework enables researchers to capture time-dependent and condition-specific gene regulation, especially in complex biological systems. Moreover, researchers have begun incorporating GRN topology to address dynamic changes in transcription. ProTINA (Protein Target Inference by Network Analysis) was developed to evaluate drug-induced network dysregulation by combining dynamic protein-gene regulatory network (PGRN) models with steady-state and time-series data [163]. ProTINA calculates drug effects on protein-regulated gene activation or pathway inhibition, effectively inferring drug targets even when target gene expression changes are minimal.

Target prediction methods that integrate gene expression profiles with GRNs have evolved from static network analysis to dynamic modeling, as shown in Fig. 2. This integration enhances the ability to differentiate directly responsive targets from indirectly responsive ones [164]. However, challenges persist, including underdetermined network inference, complexity in time-series data processing, and uncertainties in network models. Future research should focus on developing accurate network inference algorithms and effectively incorporating time-series data to enhance prediction accuracy and biological insight.

#### Protein–protein interaction network integration for target identification

**Figure 3:**
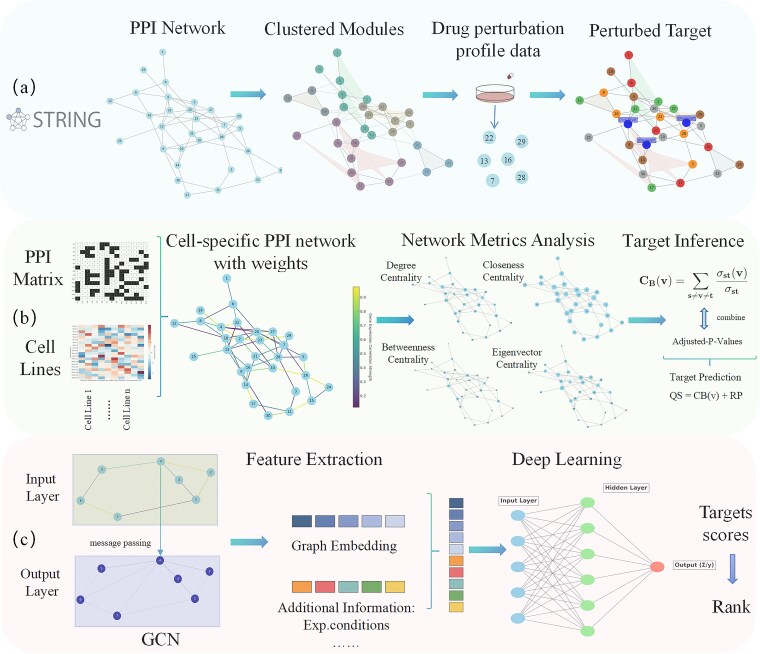
Advancements in network-based target prediction using gene expression and protein interaction data. (a) Gene expression and PPI network integration: While gene expression data provide insights into drug-induced or disease-related expression changes across the genome, their predictive power is limited by low mRNA-protein correlation, particularly in drug modulation. Integrating protein–protein interaction (PPI) networks enhances mapping of physical and functional protein associations for improved target prediction accuracy. (b) Dynamic PGRN modeling: Integrating time-dependent PPI data and network centrality metrics with gene expression data to capture dynamic regulatory changes, thereby enhancing accuracy in drug target inference. (c) Graph representation learning: Traditional ML models focus on local node information, missing broader network interactions. Graph representation learning captures high dimensional relationships across biological networks, enhancing precision and comprehensiveness in target prediction.

Gene expression data offer a comprehensive view of genome-wide expression changes under drug treatment or disease states, but relying solely on these data has limitations due to low correlation between mRNA and protein levels, which may not accurately reflect functional protein changes, especially in drug modulation contexts [165, 166]. To address this, researchers have incorporated PPI networks, which map physical and functional protein associations, with gene expression data [167], as shown in Fig. 3a. This integrated approach enhances drug target identification. For example, Ma *et al.* [168] analyzed drug-induced gene expression changes in breast cancer cell lines using PPI networks and clustering algorithms, like Walktrap, revealing that integrating PPI networks with gene expression improves target prediction accuracy. Similarly, the Herb-CMap project used heat diffusion algorithms with PPI networks and herb-induced gene expression changes to predict herbal targets, demonstrating the potential of PPI networks for complex drug systems [169].

Many existing studies primarily rely on static network properties, which limits their ability to account for dynamic changes in protein-gene regulation induced by drug interactions (Fig. 3b). Addressing this, the ProTINA algorithm introduces a dynamic PGRN model for enhanced target prediction [163]. Similarly, the TREAP approach, proposed in another study, integrates insights from network topology and gene regulation by combining centrality values and gene expression data from both PPI and protein–gene interaction networks to infer drug targets [164]. Another challenge is indirect correlations in biological networks where drug treatments affect not only target proteins but also upstream and downstream genes. This indirect effect can lower prediction accuracy. To address this issue, some studies have incorporated direct and indirect correlation features from gene expression profiles into a random forest model, improving prediction accuracy by considering the effects of upstream and downstream genes in the target regulatory network and thereby reducing false-positive results [170].

Although ML methods have demonstrated advantages in target identification, they typically focus on local node information, making it challenging to systematically analyze relationships across the entire biological network [171]. Graph representation learning offers innovative methods for target prediction by capturing high-dimensional network structures (Fig. 3c). Zhong *et al.* [133] developed the SSGCN model, which combines PPI networks and gene expression data, capturing complex, multilevel information through graphical embedding, considering factors like compound concentration and cellular context. Unlike traditional QSAR methods, the SSGCN model works well for drug–target interactions with low chemical or protein similarity, demonstrating strong generalization in target prediction. For instance, it identified Cyclophilin A as a target of nelfinavir and predicted methotrexate as an ENPP1 inhibitor using gene expression data from 22 425 compounds. To enable more precise drug target identification, studies have explored integrating a broader range of biomedical knowledge, such as DNA, mRNA, lncRNA, miRNA, transcription factors, and RNA-binding proteins, thereby achieving a more granular representation of post-transcriptional and post-translational regulation. For instance, PertKGE [145] disentangles compound-target interactions from perturbational transcriptomics through knowledge graph embedding, incorporating multi-layer regulatory events that share semantic context in biological systems. This approach significantly improves deconvolution accuracy in the “cold-start” scenario of novel compounds, demonstrating the key role of integrating multi-layer regulatory events in mitigating representational bias. Although GCN models show significant potential, their performance relies heavily on input network quality, and model interpretability is limited by deep learning complexity. Additionally, data sparsity and imbalance remain challenges for model convergence.

### Drug target prediction based on association analysis of large-scale gene expression profile

The similarity between compound-induced gene expression profiles and response patterns from shRNA or knockout experiments forms a theoretical foundation for association-based target prediction [172]. Notably, research has shown that in ~25% of target prediction tasks, gene expression profile-based models perform comparably to or even better than chemical fingerprint models [173]. This suggests that gene expression features offer a complementary advantage to structure-based approaches, providing a new pathway to overcome the reliance of traditional models on chemical structure similarity for inferring compound targets. With advancements in high-throughput gene expression profiling, integrating transcriptomics data with association analysis and supervised learning has become a key strategy for drug target identification [174]. This multilevel approach enhances prediction accuracy and provides deeper insights into drug mechanisms.

#### Comparative analysis

**Figure 4:**
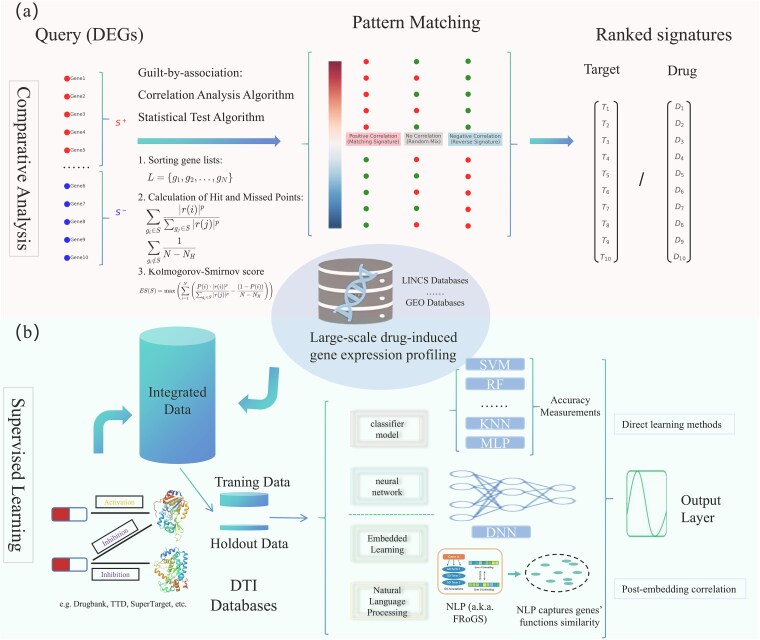
Comparative gene expression profiling and advanced predictive techniques for drug target identification. (a) Core steps in comparative profiling include constructing a reference profile, generating a query gene signature, and using algorithms to match expression patterns for target prediction. (b) ML approaches analyze gene expression data at scale, while deep learning models capture complex features to boost predictive performance. NLP techniques mitigate data sparsity issues and enhance accuracy by detecting functional gene overlaps.

Comparative gene expression profiling assumes that compounds or gene perturbations affecting the same target trigger similar downstream gene expression patterns. Known as the “guilt-by-association” theory [175], this concept has been validated in several large-scale datasets, including LINCS, which contains over 1.3 million gene expression profiles covering 22 000 genes and 20 000 pharmacological perturbations [41]. These resources are valuable for new drug discovery [176, 177], drug repositioning [178], and precision medicine research [179]. The core steps of comparative gene expression profiling include constructing a reference profile, generating a query gene signature, and applying algorithms to match expression patterns [180], as shown in Fig. 4a.

Early comparative analysis methods used efficient, straightforward strategies to process large datasets and identify similar gene expression signatures. Initial studies applied only the Pearson correlation coefficient to assess similarity. Later, Lamb *et al.* [95] developed the CMap method, which uses rank ordering and Kolmogorov–Smirnov scores via a GSEA-like algorithm, significantly broadening this method’s applications. As research advanced, more refined methods, such as Spearman’s correlation coefficient [181], Wilcoxon rank sum test [182], XSum [183], and XCos metrics [184] made similarity computations more flexible. With the LINCS project’s extension of the CMap dataset, the Weighted Connectivity Score was introduced to enhance similarity searches and correct background associations [185]. Despite improvements, these methods often lack statistical rigor at the individual reference signature level, which limits their ability to filter erroneous connections. To address these issues, tools like ZhangScore [186], ProbCMap [187], L1000CDS2 [188], and CMapBatch [123] were developed to improve similarity metrics and accuracy in drug target prediction, as analyzed in related literature [125, 178, 180, 189, 190].

Data-specific factors, including gene signature length, cell type, and batch effects, still significantly affect prediction accuracy. To address these issues, various methods have been developed to enhance comparative gene signature analysis. Traditional linear models (e.g. Limma) are unreliable with small sample sizes. Nigsch *et al.* [191] established a baseline using genetic algorithms, finding that a 128-probe set maximizes biological variation and optimizes prediction accuracy. The Characteristic Direction method further improves DEG detection by weighting both the magnitude and direction of expression changes [188]. The length of the gene signature is critical in the performance of the matching algorithm. Gene-signature progression methods optimize gene signature construction by determining minimum lengths that meet a predefined false discovery rate and by ranking genes using *P*-values and effect sizes [192]. The OneComp algorithm, developed by He *et al.* [127], enhances prediction accuracy by focusing on gene pairs with stable relative expression ordering. Additionally, the gene signature length impacts matching algorithm performance, with Lin *et al.* [125] demonstrating that ZhangScore performs optimally with signature sizes between 10 and 200. The FocusHeuristics algorithm, by Ernst *et al.* [193], improves predictive reliability by filtering out highly expressed genes and reducing genes with large fold changes. Cell type differences further complicate predictions, as a substantial proportion of perturbations show cell-type specificity. To address this, researchers have developed cross-cell lineage matching algorithms, such as SigMat [194], which improve signature matching accuracy, especially in sparse datasets. These improvements significantly enhance the accuracy and reliability of gene signature matching, providing more robust tools and strategies for drug target prediction.

#### Supervised learning

Traditional gene expression profile similarity prediction methods lack robustness and often struggle to capture biologically meaningful similarities accurately. To address these challenges, researchers are increasingly utilizing advanced supervised learning methods to enhance predictive accuracy by leveraging detailed information from gene expression data. ML advancements have provided precise solutions for drug target prediction on a large scale (Fig. 4b). Bundy *et al.* [134] integrated high-confidence chemical-target annotations from RefChemDB with LINCS L1000 gene expression profiles to construct separate binary classifiers for 51 specific targets, aimed at predicting whether a compound acts on those targets. The study evaluated the performance of multiple ML algorithms, including support vector machines, multilayer perceptrons, naïve Bayes, and k-nearest neighbors. These models, trained on individual targets, demonstrated high classification accuracy in internal cross-validation, with scores ranging approximately from 0.73 to 0.94, while accuracy on an independent hold-out test set varied between 0.68 and 0.92. However, model performance depends on the specific cellular environment, suggesting further optimization and validation are required for broader generalizability across cell types.

Deep learning further enhances drug target prediction by automatically extracting features from high-dimensional and complex data, uncovering intricate biological relationships (see Fig. 4b). Xie *et al.* [195] developed a deep neural network (DNN) model using L1000 gene expression data combined with known drug-target interactions from DrugBank. The model reduced data dimensionality to 1/200 of the original size through forward propagation, successfully establishing decision boundaries for DTIs and validating some novel DTIs. Subsequently, Lee *et al.* [136] compared various deep learning models, finding that DNN models based on L1000 data outperformed traditional ML methods, such as Naive Bayes, random forest, and logistic regression. Deep learning captures nonlinear data relationships and extracts biologically relevant features via multilayer neural networks, significantly enhancing predictive performance.

While DNNs automatically capture advanced features, model performance heavily depends on network architecture complexity. Overly complex models risk overfitting, especially when using platform-specific gene sets like the L1000, which directly measures expression levels of 978 landmark genes rather than the whole transcriptome. Additionally, deep learning models’ abstract feature representation limits interpretability, complicating result analysis. To refine drug target prediction, researchers have introduced novel embedding techniques to minimize noise in gene expression data. Filzen *et al.* [137] developed the “barcode technique,” generating perturbed barcodes by encoding gene expression profiles to capture critical biological features often masked by noise. This method, based on z-score-processed mRNA expression data and deep metric learning, enhances drug screening specificity through hash queries. Similarly, Donner *et al.* [135] developed a deep learning-based embedding algorithm to predict drug pharmacological similarity more accurately by denoising gene expression data, making replicate samples of the same compound more alike. Although further improvements in hyperparameter optimization and sample aggregation are needed, this approach shows promise in gene expression data denoising. Wei *et al.* [138] introduced the DrSim model, which optimizes gene signature similarity inference by combining principal component analysis (PCA) with linear discriminant analysis (LDA), maximizing intraclass similarity and interclass variability.

While most studies treat genes as independent functional units, often overlooking their inter-functional associations, recent advancements have applied NLP techniques to capture these associations among genes. This approach addresses data sparsity and enhances prediction accuracy. For example, FRoGS utilized a model similar to “word2vec” to encode genes as functional vectors, combining GO (gene ontology) hypergraphs with ARCHS4 experimental data to map individual human genes in a high-dimensional space, improving DTI prediction accuracy [139]. Additional gene and GO embedding methods include OPA2Vec [196], Gene2vec [197], and clusDCA [198]. By embedding gene representations and leveraging twin neural networks for training, these methods efficiently predict compound interactions with genetic perturbations in comparable biological contexts. These approaches provide powerful tools for drug repurposing and novel drug discovery, significantly enhancing prediction accuracy and efficiency.

### Integrating expression data with additional biological information for target prediction

Past research primarily focused on single-data predictions, while multimodal fusion is now recognized as a more efficient and accurate approach with the advancement of information fusion techniques. By integrating biological, topological, and physicochemical data, multimodal fusion provides a more comprehensive understanding of biological mechanisms [18]. Fusion techniques applied to expression data include early and late fusion (Table 4). Early fusion combines multiple data types (e.g. gene expression, chemical structure, and cellular imaging data) into a single dataset during preprocessing for integrated analysis. In contrast, late fusion processes each data type independently, merging the results only at the final stage. While early fusion is straightforward and often simpler, late fusion has shown to improve model robustness and accuracy in many studies by minimizing noise from individual data sources [18].

**Table 4:** Characteristics of data fusion methods.

Fusion strategy	Early fusion/feature-level fusion	Late fusion/decision-level fusion
Integration strategy	Combining different data types through simple methods like concatenation or weighted averages into a single dataset. Suitable for early-stage integration of various data types.	After each data type is processed independently by its respective model, the model outputs are then combined using methods such as selective weighting, decision voting, or weighted modality.
Advantages	This approach allows information sharing and enables the direct integration of multimodal data, ideal for scenarios requiring multi-dimensional data fusion. High flexibility in unifying various data sources.	Independently processes each modality, reducing noise from a single source and improving model robustness, thus enhancing prediction accuracy.
Disadvantages	May encounter dimensionality challenges, and complex interactions between modalities are difficult to capture.	Requires multiple models, leading to higher computational costs. Amplified noise from individual data sources may affect the final results.
Complexity	Simple in terms of computation but requires extensive preprocessing of different data types.	Higher computational complexity is needed to manage and process different data modalities independently.
Application scenarios	Suitable for direct fusion of various modalities, especially when direct interaction between modalities is needed, such as in biological, chemical, and gene expression interactions.	Ideal for integrating information after independent processing of data sources, especially in scenarios where modalities are imbalanced or multiple data sources are required for decision-making.
Representative methods	Tri-modal Hidden Markov Model (THMM), Support Vector Machine (SVM), Early Fusion LSTM, Multiple Kernel Learning (MKL)	Averaging, Deep Fusion (DF), Select-Additive Learning CNN (SAL-CNN), Majority Voting
Information interaction	Directly integrates all features, with the model automatically learning the relationships between modalities.	Modality data is processed independently, lacking low-level information interaction.
Interpretability	Difficult to distinguish the specific contribution of each modality to the final outcome.	Strong interpretability, as the independent performance of each modality’s model can be analyzed.
Computational complexity	Only one model needs to be trained, but complexity increases with high-dimensional data.	Each modality requires its own model, resulting in higher computational costs.

**Figure 5:**
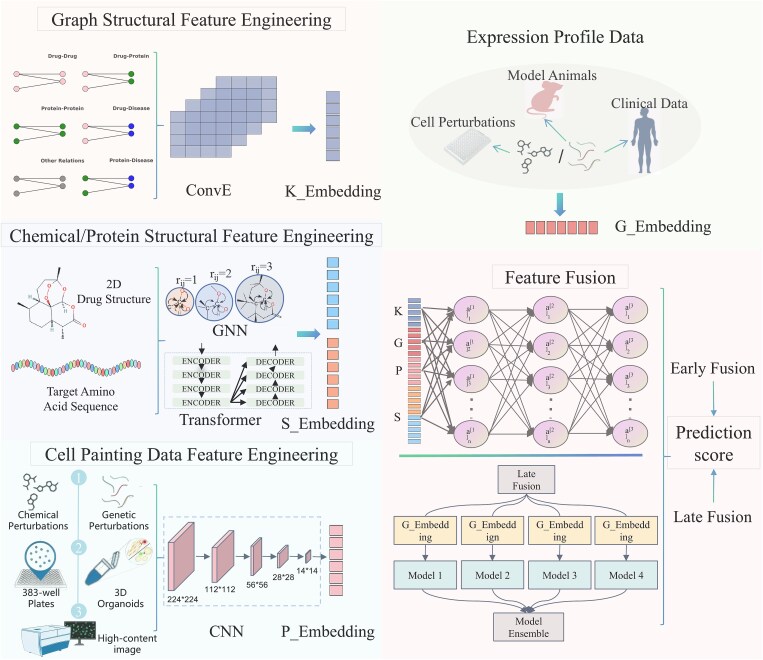
Multimodal data fusion enhances the accuracy of drug target prediction. A common approach involves combining large-scale drug-induced gene expression profiles with multiple data types, such as structural data and biological networks, through model training. By utilizing drug–target relationships from public databases as labels, deep learning models are employed to predict the potential targets of unknown compounds.

In multimodal data fusion for drug discovery, integration strategies are typically categorized by stage into early (feature-level) and late (decision-level) fusion. This section elaborates on the specific mechanisms employed by representative models to illustrate these approaches. A prevalent paradigm involves training models on large-scale drug-induced gene expression profiles alongside other data types, using known drug–target interactions as labels to predict targets for novel compounds (Fig. 5). Physicochemical properties, such as chemical structures, are widely utilized. For instance, BANDIT integrates over 20 million data points within a Bayesian framework, including information from six sources: drug efficacy, transcriptional responses, chemical structures, adverse reactions, bioassay outcomes, and known targets [140]. Its core mechanism is a Bayesian likelihood ratio framework: pairwise drug similarity scores from each source are converted into independent likelihood ratios, which are combined into an aggregate score proportional to the odds of two drugs sharing a target. Following a similar multi-source paradigm, models like FMBS [144], MoAble [141], and ceSAR [27] also enhance target prediction, providing valuable computational tools for drug discovery.

Other models employ distinct, structured architectures for feature-level fusion. The MDTips framework [142], e.g. processes different modalities with specialized encoders: Attentive FP for drug molecular graphs, Transformer for target protein sequences, fully connected networks for gene expression signatures, and ConvE for knowledge graph embeddings. These feature vectors are then concatenated and fed into a final predictor. Similarly, GraphDTI [143] constructs local subgraphs centered on target proteins from PPI networks, combining this topological information with drug structures (Mol2vec), protein sequences (ProtVec), and binding site data (Bionoi autoencoder). To ensure robustness, it employs permutation-based feature selection and cluster-based data splitting to mitigate overfitting. For complex systems like natural products, the SETComp model [146] proposes a dedicated scheme. It uses separate encoders for compounds (GNNs), genes (protein language models & PPI networks), and cellular states [variational autoencoder (VAE)], integrates them via a set embedding module (Deep Sets & Set Transformer) to achieve permutation invariance, and employs a two-stage transfer learning strategy.

In terms of integrating cell painting data, Way *et al.* [20] demonstrated how combining gene expression and cell morphology data, using unsupervised matching and supervised deep learning models, can enhance predictions of MOA and gene targets. This approach is complementary, as these datasets capture partially overlapping yet distinct mechanisms. Moshkov *et al.* [18] further showed that integrating chemical structure, imaging, and gene expression data improves compound activity prediction accuracy by a factor of two to three over unimodal models, highlighting the value of multimodal data in DTI prediction. Some studies have integrated induced transcriptome perturbations and cellular morphology data into biological networks, constructing chemical–gene–pathway–morphological and disease relationships to assess chemical-phenotypic associations [199]. Despite these advancements, the integration of large-scale multi-omics and imaging data remains challenging. Current models aim to combine all data modalities to accurately predict biological outcomes, though some modalities introduce noise that affects model accuracy. Late fusion methods, which handle each data modality independently, often outperform early fusion by reducing noise from uncorrelated data sources [18].

## Conclusions and future directions

Drug target discovery remains central to drug development, but traditional computational and experimental methods face clear limitations. Approaches like molecular docking and QSAR modeling, which depend on known protein structures or chemical information, struggle to predict unknown or complex targets effectively. Molecular docking, for instance, requires high-quality protein structural data and often lacks scoring accuracy, limiting its use in large-scale genome-wide screening. Experimental target validation methods, such as labeling techniques, are specific yet costly and time-intensive, making them inefficient for handling large compound libraries. These challenges intensify in studying complex diseases (e.g. cancer, neurological disorders), where unclear molecular mechanisms hinder target identification, reducing the efficacy of traditional methods. Consequently, new computational strategies have emerged as critical solutions. This paper reviews target prediction methods based on drug-induced transcriptomic data, highlighting the advantages over traditional computational approaches. We examine computational strategies, including gene expression profile association analysis, GCN and PPI network integration, and multimodal methods. Advances in ML, particularly deep learning, are discussed for their ability to reveal biological relationships in large-scale data and enhance predictive accuracy. We outline key factors making transcriptomic data-based prediction a valuable approach for drug target identification.

Data integration and deep learning: Future research should prioritize enhanced multimodal data integration, combining diverse sources such as single-cell transcriptomics, cellular mapping, and proteomics to improve analytical accuracy and comprehensiveness [40, 200, 201]. Deep learning algorithms, such as graph convolutional networks and variational autoencoders, allow extraction of relevant features from complex data [202]. Researchers should focus on developing hybrid models that combine the efficiency of traditional ML with the advanced pattern recognition of deep learning, especially where data scarcity or sample bias is present [203]. Additionally, interpretable AI models, using attention mechanisms to improve biological validation, should be prioritized to increase transparency and reliability [204].

Noise Reduction: High levels of noise in gene expression data present a significant challenge to prediction accuracy in model-based analyses [205, 206]. Future development will focus on optimizing denoising methods. For example, the TranSiGen model developed by Tong *et al.* [71] utilizes a VAE framework combined with self-supervised representation learning to remove confounding factors and noise from the expression spectrum, thereby improving the robustness and accuracy of the model and demonstrating the potential for development in this field. Future research can combine emerging deep learning technologies such as Generative Adversarial Networks to maintain the biological characteristics of data and improve noise processing capabilities in high-dimensional data and with insufficient sample sizes [207, 208].

Multi-platform integration and standardization of data: In order to improve the wide applicability of target prediction, it is necessary to construct a consortium level reference transcriptome through large-scale integration of public data sources (such as GEO, ArrayExpress, Human Protein Atlas, etc.) in the future. This integration will help overcome data heterogeneity issues between platforms and technologies, and enhance the predictive accuracy of the model [209]. Therefore, it is crucial to develop standardized protocols and computational methods to coordinate data from different platforms, in order to achieve higher quality data integration and support the application of target prediction models.

Integration of single-cell and spatial transcriptome data: Although traditional gene expression data mainly comes from mixed cell samples, future research can provide higher resolution cell specific data through single-cell sequencing technology and spatial transcriptomics [210, 211]. These data can reveal the different reactions of drugs in different cells, thereby more accurately predicting the multi-target effects of drugs. This combination not only improves target recognition, but also provides additional support for personalized therapy and precision medicine [212].

In conclusion, we believe that the integration of phenotypic screening and computational methods, especially in combination with drug-induced gene expression profiling and multimodal data analysis, provides a new path for the prediction of drug multi-target interactions. These technological advances will help to complement the shortcomings of the current target prediction methods based on ligand docking or experimental approaches. Although there is still room for improvement in data integration, algorithm optimization, and the accuracy of target identification, we are expected to gain a deeper understanding of the mechanism of action of drugs in this way, especially in complex diseases, with the improvement of the data analysis methods and computational power.

**Key Points**

- Reviews computational models using large-scale gene expression for DTI prediction.
- Categorizes methods: network-based, association-based, and multimodal.
- Facilitates data-driven DTI discovery with minimal reliance on prior target knowledge.
- Proposes future research focus: multi-omics integration & algorithm optimization.

## Data Availability

No datasets have been utilized in this review paper.
